# Circulating granulocyte colony‐stimulating factor as a sex‐unbiased global marker of lung and colorectal cancer cachexia pre‐clinical models

**DOI:** 10.1113/EP093809

**Published:** 2026-08-13

**Authors:** Francielly Morena, Eleanor R. Schrems, Ana Regina Cabrera, Ruqaiza Muhyudin, Sepideh Shakeri, Stavroula Tsitkanou, Mark Viggars, Tyrone A. Washington, Nicholas P. Greene

**Affiliations:** ^1^ Cachexia Research Laboratory, Exercise Science Research Center, Department of Health, Human Performance and Recreation University of Arkansas Fayetteville Arkansas USA; ^2^ Exercise Muscle Biology Laboratory, Exercise Science Research Center, Department of Health, Human Performance and Recreation University of Arkansas Fayetteville Arkansas USA; ^3^ Department of Physiology and Aging, College of Medicine University of Florida Gainesville Florida USA

**Keywords:** atrophy, cancer cachexia, chemokine, cytokine, inflammation, RNA sequencing, skeletal muscle

## Abstract

Cancer cachexia, affecting up to 80% of patients with advanced cancer, is characterized by metabolic and inflammatory dysregulation driven by tumour‐ and host‐derived factors. Although cytokines are central to cachexia pathogenesis, their circulating profiles and transcriptional relevance across models and sexes remain incompletely defined. We characterized plasma cytokines and skeletal muscle inflammatory signatures in multiple cachexia models, including Lewis Lung Carcinoma (LLC), Colon‐26 (C26) and *Apc^Min/+^* mice. Animals were monitored for 4 weeks (LLC), 25 days (C26) or until ∼20 weeks of age (*Apc^Min/+^*) per model appropriate endpoints. Publicly available RNA‐sequencing datasets from gastrocnemius (LLC) and tibialis anterior (C26 and *Apc^Min/+^*) muscles were integrated with plasma profiling using a Mouse Cytokine/Chemokine 32‐Plex assay. To enhance accessibility and reproducibility, we developed an interactive Shiny application – thecachexiatlas/Cytokine Explorer – enabling dynamic visualization of cytokine levels, transcriptional signatures and phenotype correlations across models and sexes. Cytokine receptor encoding genes (*Il6ra*, *Il4ra*, C*sf3*, *Osmr*) and inflammatory pathways were consistently enriched in skeletal muscle of cachectic animals across models and sexes. Among circulating cytokines, granulocyte colony‐stimulating factor (G‐CSF) was uniquely elevated in all models and both sexes. Elevated G‐CSF levels exhibited stronger negative correlations with tibialis anterior muscle mass than the commonly used cytokine interleukin (IL)‐6, particularly when expressed as the ratio of G‐CSF to the anti‐inflammatory cytokine IL‐13. The G‐CSF:IL‐13 ratio may represent a robust global biomarker of cachexia severity. The Cytokine Explorer Shiny app provides an open, interactive platform to facilitate hypothesis generation and advance research in cancer cachexia.

## Abstract (highlights)

- **What is the central question of this study?**
  What are the plasma cytokine profiles of male and female mice in models of lung and colorectal cancers?
- **What is the main finding and its importance?**
  The Cytokine Explorer Shiny app was created, providing an open, interactive platform to explore the relationship between 32 cytokines and several phenotypes in three models of cachexia and both sexes. Elevated granulocyte colony‐stimulating factor (G‐CSF) levels exhibited stronger and sex‐unbiased negative correlations with tibialis anterior muscle mass than the commonly used cytokine interleukin (IL)‐6, particularly when expressed as the ratio of G‐CSF to the anti‐inflammatory cytokine IL‐13.

## 1 INTRODUCTION

Cancer cachexia is a complex, multifactorial syndrome characterized by severe body weight loss, muscle atrophy and systemic inflammation (Fearon et al., 2013), often leading to decreased quality of life and reduced survival rates in cancer patients. Cachexia, which affects up to 80% of patients with advanced cancer (Argilés et al., 2015), is characterized by complex metabolic and inflammatory alterations influenced by tumour‐derived factors, as well as host‐derived factors including impaired lipid metabolism and insulin resistance (Kim et al., 2012; McNamara et al., 1992). The pathophysiological mechanisms underlying cachexia involve a network of pro‐inflammatory cytokines, metabolic dysregulation and alterations in muscle protein turnover, together contributing to the wasting phenotype observed in affected individuals (Argilés et al., 2015; Fearon et al., 2011).

Cytokines such as tumour necrosis factor‐α (TNF‐α), interleukin (IL)‐6 and IL‐1β are central to the inflammatory response associated with cachexia (Clamon et al., 2022). When chronically elevated, these cytokines are known to promote muscle protein degradation and inhibit protein synthesis through various signalling pathways, including the ubiquitin–proteasome system and autophagy (Sandri, 2013). Elevated levels of TNF‐α, IL‐6 and other pro‐inflammatory cytokines have been consistently observed in clinical conditions and preclinical models of cachexia, underscoring their pivotal role in this syndrome (Kim et al., 2012; McNamara et al., 1992). The role of cytokines in the pathogenesis of cachexia has been a focal point of research. TNF‐α, once known as cachectin, induces cachexia‐like symptoms when administered to animals, including anorexia, weight loss and muscle wasting (McNamara et al., 1992). Studies have further demonstrated that TNF‐α can stimulate lipolysis and inhibit lipoprotein lipase activity, contributing to fat depletion and the characteristic weight loss seen in cachexia (Beutler & Cerami, 1986). IL‐6 is a key driver of cachexia in various cancer models (Baltgalvis et al., 2008; Hetzler et al., 2015). Cachexia in colorectal and lung cancer‐bearing male mice appears dependent on high levels of IL‐6 expression and activation of its downstream atrophic signalling target, signal transducer and activator of transcription 3 (STAT3), in the skeletal muscle (Bonetto et al., 2012; White et al., 2013). Along with effects on the skeletal muscle, circulating IL‐6 also has implications for cachexia development, where high plasma IL‐6 concentrations are associated with increased severity of cachexia (Bonetto et al., 2012; White et al., 2013). Understanding the roles of these cytokines in cachexia is essential to identify potential targets of therapeutic treatment to mitigate muscle wasting.

Many current models of cachexia exist across various types of cancer. Colorectal and lung cancer have some of the highest incidences of cachexia, affecting approximately 40–50% of patients (Mariean et al., 2023). Therefore, understanding mechanisms associated with cachexia among these cancers is critical to enhance patient outcomes. Furthermore, research has brought to light the importance of understanding biological sex differences and their associations with the multifactorial metabolic dysfunction in these conditions (Satari et al., 2025). Our laboratory, among others, demonstrated implications of biological sex differences in the cachectic responses among lung and colorectal cancer. Specifically, we have demonstrated female mice inoculated with Lewis Lung Carcinoma (LLC) or with Colon‐26 carcinoma cell (C26) tumour allografts display a delayed and/or mitigated muscle phenotype compared with males (Cabrera et al., 2023; Lim et al., 2022; Morena Da Silva et al., 2023). Finally, in the genetic *Apc^Min/+^* model of colorectal cancer, we have also observed exacerbated morbidity in male compared with female mice (Schrems et al., 2023). Findings by other investigators have demonstrated cachexia in the *Apc^Min/+^* model to be IL‐6 dependent in male, but not in female mice (Hetzler et al., 2015). Recent research in pancreatic cancer cachexia has similarly shown males to experience earlier onset of muscle loss in cachexia compared with females (Zhong et al., 2022). Together, these findings highlight the importance of understanding the multifactorial nature of cachexia as it encapsulates biological sex. Despite the growing evidence for sex‐specific differences in cachexia, systematic comparisons of circulating cytokine profiles across multiple cancer cachexia models remain underexplored. A deeper understanding of differences in the secretome and consequences of changes in circulating factors is required to develop more effective treatments of the condition.

We hypothesize that cytokine profiles would be largely cancer and sex specific, while common cytokine response to tumour burden could be representative of cachectic phenotype and severity. Therefore, we aimed to delineate the plasma cytokine profiles of male and female mice in models of lung and colorectal cancers. To do so, mice bearing LLC or C26 tumours, as well as *Apc^Min/+^* mice, were used with a 32‐analyte multiplex cytokine/chemokine analysis. By quantifying key cytokines, we seek to elucidate their potential roles in the pathogenesis of cachexia with consideration of both biological sexes. Understanding cytokine dynamics provides valuable insights into the molecular mechanisms driving cachexia and may lead to the identification of potential biomarkers and therapeutic targets.

## 2 METHODS

### 2.1 Ethical approval

Animal experiments were ethically conducted and approved by the University of Arkansas Institutional Animal Care and Use Committee (IACUC; protocol IDs: 22004, 23007, 20041, 15065), and were in accordance with *Experimental Physiology*’s policies regarding animal experiments.

### 2.2 Animals and housing

Animals were kept on a 12:12 light–dark cycle and given ad libitum access to food and water. Animals for this study are selected cohorts from prior studies (Brown et al., 2018; Lim et al., 2022; Schrems et al., 2023). Male and female C57BL/6J (Stock No.: 000664), BALB/c (Stock No.: 001026) and male *Apc^Min/+^* (ApcMin/+: IMSR_JAX:002020) mice were purchased from The Jackson Laboratory (Bar Harbor, ME, USA).

Induction of LLC was done as described by Lim et al. (2022). Briefly, for induction of the LLC model, male and female C57BL/6J mice were inoculated with LLC cells at 8 weeks of age by injection bilaterally to each hind flank. The cell solution consisted of 1 × 10^6^ cells suspended in 200 µL of sterile phosphate buffered saline (PBS). An equal volume of PBS was injected to the left hind flank for control animals. Tumours were allowed to develop for up to 4 weeks.

Tumour inoculation and induction of C26 was done as described by Cabrera et al. (2023). For the C26 model of cachexia, male and female BALB/c mice were inoculated with Colon‐26 Carcinoma cells (C26, National Cancer Institute, Frederick, MD, USA, originally derived from female BALB/c mouse cells [1149045]) at 8 weeks of age. Cell injections were 5 × 10^5^ cells to each side, for a total of 1 × 10^6^ cells. Cells were suspended in 100 µL of sterile PBS or equal volume of sterile PBS as control. Tumours were allowed to develop for 25 days where cachexia phenotype is mild.

Finally, for the *Apc^Min/+^* (APC) model, APC male mice were bred with female C57Bl/6J mice. At 4 weeks of age, mice were weaned and genotyped as described (Schrems et al., 2023). Briefly, DNA was isolated from tail or ear snips using semi‐quantitative PCR for identification of the *Apc^Min/+^* gene. Forward primer (GGG AAG TTT AGA CAG TTC TCG T) and reverse primer (TGT TGG ATG GTA AGC ACT GAG) were used. Mice of ∼20 weeks of age, per model appropriate humane endpoints, were used to represent a severe cachectic phenotype.

At experimental endpoint for each model, animals were humanely euthanized via cardiac puncture exsanguination under deep oxygen mixed isoflurane anesthesia (3–5%, flow rate 1 L/min). Blood was collected and tissues dissected, weighed, snap‐frozen in liquid nitrogen and stored at −80°C for further analysis. Phenotypic characteristics of cachexia have been previously published by our laboratory as parts of larger experiments (Brown et al., 2017; Lim et al., 2022; Morena et al., 2024) as well as the phenotype from the RNA‐sequencing subset (Morena et al., 2024). Table 1 describes the complete phenotype relevant subset of animals presented here.

**TABLE 1:** Phenotypic characterization of cachexia.

	Females		Males		Significance	*P*
**Model: LLC**						
**Group**	PBS (*n* = 8)	LLC (*n* = 8)	PBS (*n* = 8)	LLC (*n* = 8)		
**Body weight (g)**	18.94 ± 0.21	21.33 ± 0.56	26.66 ± 0.51	26.23 ± 0.61	Interaction	0.0086
**Tumour weight (g)**	N/A	3.04 ± 0.17	N/A	1.23 ± 0.24	*	<0.0001
**Body weight—tumour weight (g)**	18.94 ± 0.21	18.29 ± 0.55	26.66 ± 0.51	25 ± 0.62	Sex + Cancer	<0.0001, 0.028
**Gastrocnemius (mg/mm)**	5.47 ± 0.09	5.32 ± 0.08	8.11 ± 0.18	7.87 ± 0.16	Sex	<0.0001
**TA (mg/mm)**	2.22 ± 0.04	1.95 ± 0.06	2.68 ± 0.06	2.78 ± 0.04	Interaction	0.0011
**Soleus (mg/mm)**	0.45 ± 0.01	0.36 ± 0.02	0.57 ± 0.02	0.52 ± 0.02	Sex + Cancer	<0.0001, 0.0002
**Plantaris (mg/mm)**	0.78 ± 0.02	0.73 ± 0.02	1.14 ± 0.04	1.12 ± 0.04	Sex	<0.0001
**EDL (mg/mm)**	0.50 ± 0.01	0.45 ± 0.01	0.57 ± 0.03	0.58 ± 0.04	Sex	0.0004
**Fat (mg)**	321.8 ± 30.8	162.8 ± 27.80	460.8 ± 61.9	354.8 ± 38.9	Sex + Cancer	0.0005, 0.0036
**Spleen (mg)**	80.3 ± 4.95	333.7 ± 27.25	74.5 ± 3.36	172.91 ± 23.1	Interaction	0.0002
**Liver (mg)**	727.4 ± 30.27	1053.3 ± 46.38	1237.1 ± 57.00	1156.2 ± 66.5	Interaction	0.0005
**Heart (mg)**	105.1 ± 3.81	105.9 ± 3.74	125.6 ± 3.38	123.5 ± 4.56	Sex	<0.0001
**Tibia (mm)**	16.42 ± 0.12	16.57 ± 0.05	17.19 ± 0.16	17.10 ± 0.18	Sex	<0.0001
**Model: C26**						
**Group**	PBS (*n* = 8)	C26 (*n* = 8)	PBS (*n* = 8)	C26 (*n* = 8)		
**Body mass (g)**	21.67 ± 0.42	22.01 ± 0.42	27.4 ± 0.53	25.87 ± 0.67	Sex	<0.0001
**Tumour mass (g)**	N/A	0.96 ± 0.19	N/A	2.46 ± 0.25	Interact	0.0003
**Tumour‐free body mass (g)**	21.67 ± 0.42	21.1 ± 0.62	27.4 ± 0.53	23.33 ± 0.58	Interaction	0.0033
**Gastrocnemius (mg/mm)**	6.33 ± 0.10	6.28 ± 0.13	8.27 ± 0.11	7.22 ± 0.25	Interaction	0.0293
**TA (mg/mm)**	2.42 ± 0.04	2.25 ± 0.11	2.77 ± 0.07	2.45 ± 0.08	Sex + Cancer	0.0018, 0.0051
**Soleus (mg/mm)**	0.49 ± 0.01	0.46 ± 0.01	0.55 ± 0.03	0.51 ± 0.01	Sex	0.0068
**Plantaris (mg/mm)**	0.91 ± 0.01	0.91 ± 0.04	1.23 ± 0.06	1.12 ± 0.04	Sex	<0.0001
**EDL (mg/mm)**	0.58 ± 0.03	0.53 ± 0.02	0.62 ± 0.05	0.55 ± 0.02	ns	ns
**Fat (mg)**	8.01 ± 0.21	7.69 ± 0.20	28.57 ± 1.51	13.93 ± 1.98	Interaction	0.0477
**Spleen (mg)**	5.94 ± 0.15	11.89 ± 0.52	5.73 ± 0.13	14.82 ± 0.85	Cancer	<0.0001
**Liver (mg)**	64.39 ± 1.25	72.97 ± 1.34	81.48 ± 2.29	82.59 ± 2.96	Interaction	0.0377
**Heart (mg)**	8.01 ± 0.21	7.69 ± 0.20	9.38 ± 0.19	8.44 ± 0.22	Interaction	0.0409
**Tibia (mm)**	15.76 ± 0.10	15.97 ± 0.12	16.46 ± 0.08	16.27 ± 0.14	Sex	0.0002
**Model: APC**						
**Group**	WT (*n* = 8)	APC (*n* = 7)	WT (*n* = 8)	APC (*n* = 8)		
**Body weight**	23.56 ± 1.15	17.51 ± 0.5	28.15 ± 0.54	21.28 ± 0.74	Sex + Cancer	<0.0001, <0.0001
**Number of polyps**	NA	36.63 ± 4.74	NA	34.5 ± 3.81	ns	ns
**Gastrocnemius (mg/mm)**	6.75 ± 0.12	5.12 ± 0.18	8.43 ± 0.25	6.05 ± 0.54	Sex + Cancer	0.0006, <0.0001
**TA (mg/mm)**	2.32 ± 0.08	1.62 ± 0.11	2.90 ± 0.05	2.03 ± 0.13	Sex + Cancer	<0.0001
**Soleus (mg/mm)**	0.47 ± 0.02	0.41 ± 0.02	0.62 ± 0.03	0.48 ± 0.02	Sex + Cancer	<0.0001
**Plantaris (mg/mm)**	0.90 ± 0.03	0.64 ± 0.02	1.24 ± 0.04	0.85 ± 0.05	Sex + Cancer	<0.0001
**EDL (mg/mm)**	0.59 ± 0.02	0.44 ± 0.02	0.68 ± 0.04	0.49 ± 0.02	Sex + Cancer	0.0133, <0.0001
**Fat (mg)**	380.47 ± 55.51	87.72 ± 37.73	661.93 ± 81.0	77.53 ± 21.8	Interaction	0.0163
**Spleen (mg)**	88.41 ± 6.85	304.56 ± 41.49	73.98 ± 4.5	380.35 ± 37.8	Cancer	<0.0001
**Liver (mg)**	1013.3 ± 48.76	1016 ± 61.2	1294.97 ± 51.8	1300.9 ± 63.4	Sex	<0.0001
**Heart (mg)**	107.59 ± 5.20	106.28 ± 8.3	133.4 ± 4.79	117.71 ± 6.17	Sex	0.0068
**Tibia (mm)**	17.61 ± 0.32	16.78 ± 0.22	17.52 ± 0.09	17.21 ± 0.31	Cancer	0.0321

*Note*: ^*^Indicate T‐test significance between sex in tumor burden.

Abbreviations: EDL, extensor digitorum longus; ns, not significant; N/A, not available; TA, tibialis anterior.

### 2.3 Data visualization and analysis of RNA sequencing

Gastrocnemius (LLC), and tibialis anterior (TA) (C26 and APC) muscles were utilized for sequencing purposes. Sequencing data from these sample sets has been previously published (Morena et al., 2024), and genomic data are publicly available (GSE114820, GSE222317, GSE276018). A pre‐alignment QA/QC read was performed to assure quality, followed by a STAR 2.7.8a index alignment – RefSeq: *Mus musculus* (mouse) – mm39 GeneBank assembly. QA/QC was repeated post‐alignment. Normalization was performed by using the median ratio for DESeq2 in Partek. A filter of 30 minimum reads across all samples was applied. DESeq2 analysis used comparisons of LLC, C26 or APC against model matched control (PBS or wild‐type [WT]). Results were downloaded in text format and further organized in Excel. Cutoffs for differentially expressed genes (DEGs) were performed at 0.05 false discovery rate (FDR‐adj. *P*‐value). Pathway analysis was performed using ShinyGO 0.77, utilizing the Kyoto Encyclopedia of Genes and Genomes (KEGG) pathway and a background reference gene list comprising all genes in our dataset. RNA sequencing and pathway analysis data summaries can be found in Supporting information File 1.

### 2.4 Sample preparation for multiplex

At experimental endpoint, blood was collected from cardiac puncture and subsequently centrifuged at 17,800 G for 15 min to isolate plasma and stored at −80°C for further analysis. A Mouse Cytokine/Chemokine 32‐Plex Discovery Assay Array (MD32) was quantified in 2‐fold PBS diluted plasmas by Eve Technologies Corporation (Calgary, AB, Canada). Of the 32 cytokines analysed, 29 cytokine concentrations were within quantifiable limits (pg/mL) and examined for every downstream analysis.

### 2.5 Statistical analyses

Outlier removal was performed prior to any further analysis using the non‐parametric method, Tukey's outlier detection. This model removes points that fall far outside the central 50% of a dataset (fences). Fences were defined by Q_1_ – 1.5 × IQR and Q_3_ + 1.5 × IQR (where IQR is the interquartile range).

A sensitivity power analysis was performed using the pwr package in R. For two‐group comparisons with *n* = 7–8 per group, α = 0.05 and 80% power, the study was powered to detect large effects of approximately Cohen's *d* = 1.5–1.6. Therefore, the study was sufficiently powered to detect robust cachexia‐associated phenotypic changes, while smaller cytokine‐level differences were interpreted with caution.

A two‐way ANOVA was used for the global analysis for all phenotypic measures, excluding tumour weight, polyp number and tibia length (PBS/WT vs. Cancer [LLC, C26 and APC]). Tumour weight, polyp number, and tibia length were analysed between biological sex (male vs. female) using Student's unpaired *t*‐test. Data from RNA sequencing was analysed with DESeq2 using Partek Flow Software. Significant DEGs were defined using FDR (reported FDR step‐up adjusted *P*‐value) that was less than 0.05. For analysis of cytokine profiles, a series of pre‐planned, unpaired *t*‐tests were performed to establish the overall cytokine profile within each model and sex (PBS/WT) and cancer groups at their endpoints: LLC (4 weeks), C26 (25 days) and APC (20 weeks). To further elucidate the nature of the cytokine profile in cachexia and the interactions between biological sex and cancer model, we conducted additional analyses. First, to evaluate the role of biological sex in cancer cachexia, we performed *t*‐tests within the LLC model, and between sex (male LLC vs. female LLC). Because multiplex cytokine analyses inherently involve evaluation of multiple analytes, FDR‐adjusted *P*‐values were additionally calculated and are provided in Supporting information File 2 as a complementary measure to aid interpretation of cytokine profile data. Primary interpretation focused on biologically consistent patterns across models and effect sizes rather than adjusted *P*‐values alone. FDR‐adjusted statistics for additional cytokine comparisons and correlation analyses can also be accessed through the CytokineExplorer application.

Next, we conducted two‐way ANOVA between colorectal models of cachexia and between both sexes to determine if there were interactions of sex and cachexia severity (C26 – mild cachexia; APC – severe cachexia) in cytokine profiles. These analyses were done using two‐way ANOVA to establish main effects and interactions (sex [male vs. female] by cancer type [C26 vs. APC]). When significant interactions were observed, a Tukey's *post hoc* test was used to evaluate multiple comparisons. All ANOVAs and *t*‐tests were performed using GraphPad Prism 8.0 (GraphPad Software, San Diego, CA, USA).

Finally, Pearson, Spearman, and biweight midcorrelation coefficients (with associated *P*‐values) were calculated in R (v4.4.1) to assess associations between circulating cytokine concentrations and skeletal muscle mass (TA). Pearson correlation was used as the primary metric because our a priori question concerned the magnitude and direction of linear relationship between systemic cytokine levels and muscle mass, and Pearson provides an effect size that is directly interpretable and comparable across cytokines and models. Assumptions of normality for parametric analyses were assessed prior to statistical testing using GraphPad Prism. Given that cytokine concentrations can be heavily tailed, we additionally performed rank‐based (Spearman) and robust (biweight) correlations as sensitivity analyses to evaluate whether observed associations were disproportionately influenced by extreme values. As expected, some relationships were attenuated under robust methods, consistent with partial leverage from outliers. All three‐correlation metrics (including correlations with additional muscle, soleus, plantaris, gastrocnemius and extensor digitorum longus (EDL), as well as tumour burden and spleen weight) are available for interactive comparison in CytokineExplorer (ShinyApp: https://thecachexiatlas.shinyapps.io/CytokineExplorer/). Statistical significance was defined as α = 0.05. Robustness of correlation was assessed by significant using multiple correlation methods and is available in both the CytokineExplorer and Supporting information File 2.

## 3 RESULTS

Phenotype for all experimental cohorts presented in this study are in Table 1.

### 3.1 Peripheral transcriptional response suggests global inflammation activation in skeletal muscle during cancer cachexia

RNA sequencing from these animals has been reported elsewhere (Morena Da Silva et al., 2023; Morena et al., 2024). For the purposes of this study, we re‐examined the data with specific consideration for inflammatory pathways which may tie to circulating cytokines and chemokines. DEGs implicated in any inflammatory‐associated pathways related with the ‘immune system’ were filtered (highlighted by bolded borders in Supporting information File 1) and four additional inflammation pathways known to be involved in cachectic skeletal muscle were then included (highlighted in yellow in Supporting information File 1) (Berriel Diaz et al., 2024). Pathways shared across four or more of the six model/sex groups included Janus kinase (JAK)–STAT signalling, nuclear factor‐κB (NF‐κB) signalling, Th17 cell differentiation, complement and coagulation cascades, and leukocyte transendothelial migration (Figure 1a), indicating strong convergence in inflammatory and immune‐related biological processes across cachexia models despite differences in tumour type and biological sex. In contrast, comparison of genes associated with these enriched pathways revealed that most genes were model‐ and sex‐specific, with only a small subset shared across five groups. These shared genes included *Cdkn1a*, *Csf2rb*, *Csf2rb2*, *Csf3r*, *Il4ra*, *Il6ra*, *Mcl1*, *Myc*, *Osmr*, *Stat3* and *Tgfbr2* (Figure 1b).

When looking at each cancer model independently, LLC males, nine immune/inflammatory pathways were significantly enriched among the upregulated genes (Supporting information File 1). From these nine pathways, 201 DEGs appeared to be implicated in this enrichment (Figure 1c–e). In LLC females, we found 17 pathways significantly enriched among the upregulated genes (Supporting information File 1) with 213 DEGs involved in inflammation and/or immune system regulation (Figure 1c, e, f, Supporting information File 1). When comparing males and females, 54 DEGs were common to both including cytokine receptors (*Il6ra*, *Il2rg*, *Il4ra*, *Il1r1*, *Il13ra1*, C*sf3r* and *Osmr*), cell stress responder *Gadd45a*, cytokine activator *Jun* and transcription factor *Myc*, along with additional members of the JAK/STAT signalling pathway (*Stat3* and *Jak3*) (Figure 1f, Supporting information File 1). For the C26 model, in males, we found 10 pathways enriched regarding inflammation and immune regulation (Supporting information File 1) with 283 DEGs involved (Figure 1g–i). In C26 females, 14 pathways were enriched for inflammation and/or immune system regulation (Supporting information File 1) of which there were 167 DEGs associated (Figure 1g,i,j). Sixty‐six DEGs were shared between male and female C26 mice (Figure 1i, Supporting information File 1). Similar to LLC, shared upregulated genes included cytokine receptors (*Il1r2*, *Il4ra*, *Il6ra*, *Il13ra1*, *Il17ra*, *Il1r1*, C*sf3* and *Osmr*), the transcriptional factor *Myc*, members of the JAK/STAT signalling pathway (*Stat3* and *Jak3*), pro‐atrophic cytokine *Mstn* and several *Cxcl* transcripts (Figure 1g). Lastly, for APC males, only one pathway, the NF‐κB signalling pathway, was found to be enriched in which 27 DEGs were implicated (Figure 1k,l, Supporting information File 1). For APC females, the JAK/STAT signalling pathway and Th17 cell differentiation were the two pathways to be enriched by 46 DEGs, including C*sf3*, of which four genes (*Bcl2l1*, *Prkcq*, *Pias4* and *Rela*) were shared between males and females (Figure 1k,l, Supporting information File 1).

Collectively, these data demonstrate that cancer cachexia is associated with a broad activation of immune and inflammatory transcriptional programmes within skeletal muscle across multiple tumour models, with both shared and model‐specific signatures. The recurrent enrichment of cytokine receptor pathways and JAK/STAT‐associated signalling across models and sexes confirms, in our models, the well‐established idea that systemic inflammation and local transcriptional reprogramming occur in parallel during cachexia progression. While the magnitude and composition of transcriptional responses differed between models, the convergence of immune‐related pathways supports a common inflammatory framework underlying cachexia‐associated muscle remodelling. These findings provide a foundation for linking circulating cytokine profiles with peripheral transcriptional adaptations, which are further explored in subsequent analyses.

### 3.2 Systemic cytokine profiling reveals model‐ and sex‐specific inflammatory responses with consistent elevation of granulocyte colony‐stimulating factor

Next, we sought to assess cytokines that may interact with skeletal muscle to drive inflammatory responses in LLC, C26 and APC. Of the 32 cytokines and chemokines assayed, 29 reached quantifiable levels. Among those 29, 15 were pro‐inflammatory cytokines, seven were pro‐inflammatory chemokines, and seven were anti‐inflammatory cytokines and chemokines. Raw concentrations of these targets can be found in Table 2. Raw cytokine concentrations were analysed within sex and model to evaluate the effects of cancer on circulating cytokines by comparing tumour‐bearing groups to their respective healthy controls. All relevant data in this subsection below are in Table 2.

**TABLE 2:** Observed concentration of each cytokine in pg/mL within model and within sex in 3 models of CC (LLC, C26, and APC).

	Cytokine observed concentration (pg/mL)											
Cytokine	LLC model				C26 model				APC model			
	Male		Female		Male		Female		Male		Female	
	PBS (*n* = 8)	LLC (*n* = 8)	PBS (*n* = 8)	LLC (*n* = 8)	PBS (*n* = 8)	C26 (*n* = 8)	PBS (*n* = 8)	C26 (*n* = 8)	WT (*n* = 8)	APC (*n* = 8)	WT (*n* = 7)	APC (*n* = 8)
**Eotaxin**	857.9 ± 56	1061.7 ± 182	**1524.9 ± 157**	**867.1 ± 63**	2510.1 ± 290	1890.2 ± 229	1281.3 + 160	1776.2 ± 252	**1387.1 ± 159**	**615.3 ± 96**	**1473.7 ± 405**	**540.1 ± 83**
**G‐CSF**	**314.77 ± 34**	**2713.3 ± 889**	**1416.0 ± 230**	**20,731.7 ± 2492**	**140.4 ± 20**	**2907.2 ± 689**	**185.1 ± 24**	**4273.6 ± 1132**	**421.8 ± 3 5**	**2273.8 ± 737**	**358.3 ± 30**	**1280.0 ± 285**
**IFN‐γ**	2.6 ± 1	2.2 ± 0.3	**11.7 ± 3**	**4.5 ± 1**	3.8 ± 1	2.3 ± 1	2.9 ± 1	2.8 ± 1	3.2 ± 0.4	6.0 ± 2	4.7 ± 1	4.0 ± 1
**IL‐1α**	58.0 ± 7	50.0 ± 9	**74.7 ± 10**	**137.4 ± 10**	**31.1 ± 5**	**61.1 ± 11**	45.5 ± 16	45.7 ± 12	72.0 ± 14	130.1 ± 26	92.6 ± 12	81.2 ± 13
**IL‐1β**	4.0 ± 1	4.6 ± 1	7.1 ± 1	6.0 ± 1	3.6 ± 1	3.4 ± 1	5.5 ± 1	3.7 ± 1	3.9 ± 1	5.0 ± 0.4	**3.5 ± 0.2**	**5.2 ± 0.4**
**IL‐2**	2.5 ± 0.5	1.6 ± 0.5	**11.1 ± 1.1**	**5.0 ± 0.5**	3.6 ± 1.5	1.2 ± 0.1	10.8 ± 9	17.1 ± 9	2.0 ± 0.3	5.9 ± 2	7.2 ± 3	4.0 ± 0.7
**IL‐3**	0.4 ± 0.1	0.5 ± 0.1	1.1 ± 0.1	1.2 ± 0.1	1.4 ± 0.4	0.7 ± 0.1	3.9 ± 3	3.7 ± 2	0.8 ± 0.2	0.9 ± 0.1	0.7 ± 0.2	0.9 ± 0.1
**IL‐4**	0.3 ± 0.03	0.3 ± 0.02	0.7 ± 0.1	0.5 ± 0.03	0.3 ± 0.03	0.3 ± 0.02	0.6 ± 0.1	0.5 ± 0.03	0.3 ± 0.02	0.3 ± 0.03	0.4 ± 0.04	0.3 ± 0.01
**IL‐5**	3.3 ± 1	2.6 ± 1	**15.7 ± 4**	**3.3 ± 1**	1.8 ± 0.3	1.2 ± 0.1	9.7 ± 3	2.1 ± 1	4.2 ± 1	5.8 ± 1	6.2 ± 1	2.9 ± 2
**IL‐6**	**3.0 ± 1**	**7.6 ± 1**	31.1 ± 8	80.0 ± 35	**1.1 ± 0.3**	**246.5 ± 65**	**18.6 ± 9**	**166.3 ± 52**	**6.6 ± 2**	**90.9 ± 21**	**4.9 ± 1**	**18.9 ± 6**
**IL‐7**	2.0 ± 1	1.4 ± 0.3	3.0 ± 1	5.3 ± 1	26.2 ± 17	18.2 ± 13	512.4 ± 287	70.4 ± 42	2.1 ± 1	5.2 ± 2	7.9 ± 2	4.2 ± 1
**IL‐9**	21.0 ± 6	24.4 ± 7	37.5 ± 12	39.5 ± 9	32.6 ± 8	51.3 ± 8	85.1 ± 43	39.0 ± 19	21.7 ± 2	65.6 ± 20	54.9 ± 12	54.5 ± 14
**IL‐10**	6.11 ± 1	6.1 ± 1	11.1 ± 2	11.0 ± 1	40.5 ± 26	8.9 ± 4	459.8 ± 242	26.5 ± 17	**6.1 ± 1**	**18.5 ± 2**	10.4 ± 1	11.7 ± 1
**IL‐12p40**	25.5 ± 4	13.9 ± 3	**32.6 ± 5**	**13.2 ± 3**	21.1 ± 4	20.7 ± 11	43.4 ± 17	17.5 ± 4	35.3 ± 4	38.2 ± 9	59.8 ± 9	45.8 ± 5
**IL‐12p70**	16.7 ± 4	17.8 ± 4	26.5 ± 4	19.3 ± 2	11.9 ± 2	13.6 ± 4	61.9 ± 23	28.8 ± 11	11.6 ± 3	21.0 ± 4	15.7 ± 4	27.1 ± 4
**IL‐13**	229.3 ± 13	223.5 ± 27	**450.4 ± 4**	**245.1 ± 15**	320.5 ± 33	246.5 ± 25	300.9 ± 66	216.0 ± 16	**297.1 ± 30**	**154.6 ± 10**	282.8 ± 30	229.8 ± 16
**IL‐15**	47.1 ± 10	68.4 ± 10	94.6 ± 10	88.9 ± 7	287.5 ± 113	226.7 ± 101	1329.2 ± 991	311.1 ± 144	145.6 ± 18	121.8 ± 15	112.3 ± 13	156.5 ± 41
**IL‐17**	0.8 ± 0.1	0.8 ± 0.1	1.2 ± 0.2	0.7 ± 0.1	0.5 ± 0.1	0.2 ± 0.1	0.4 ± 0.1	0.3 ± 0.05	0.9 ± 0.1	1.2 ± 0.1	**0.7 ± 0.1**	**4.9 ± 0.1**
**IP‐10**	18.3 ± 3	27.1 ± 4	**115.3 ± 14**	**53.5 ± 8**	**19.2 ± 0.4**	**86.4 ± 5**	**14.2 ± 2**	**43.6 ± 3**	17.8 ± 2	24.9 ± 6	15.3 ± 1	17.5 ± 6
**KC**	**13.9 ± 2**	**59.3 ± 14**	143.3 ± 33	195.8 ± 34	**25.6 ± 9**	**170.6 ± 39**	**22.7 ± 9**	**96.2 ± 21**	11.1 ± 3	19.4 ± 3	38.1 ± 12	43.6 ± 12
**LIF**	**0.1 ± 0.1**	**1.0 ± 0.1**	0.8 ± 0.2	6.8 ± 3	22.7 ± 13	5.7 ± 3	164.1 ± 66	41.0 ± 23	0.6 ± 0.1	1.0 ± 0.2	4.6 ± 1.4	1.1 ± 0.4
**LIX**	1872.5 ± 327	731.1 ± 388	2754.6 ± 418	2060.3 ± 259	131.6 ± 17	145.9 ± 42	161.1 ± 65	640.9 ± 274	1353.7 ± 346	723.1 ± 239	839.3 ± 179	727.6 ± 146
**M‐CSF**	8.2 ± 1	6.6 ± 1	10.9 ± 0.4	11.9 ± 1	6.8 ± 1	6.2 ± 1	9.2 ± 3	9.0 ± 2	8.9 ± 1	9.32 ± 1	8.6 ± 1	12.1 ± 2
**MIG**	84.7 ± 8	67.1 ± 22	**3809.6 ± 460**	**598.0 ± 59**	110.1 ± 32	135.5 ± 13	264.9 ± 128	240.1 ± 67	**238.5 ± 39**	**79.7 ± 19**	123.1 ± 22	75.1 ± 19
**MIP‐1α**	20.9 ± 4	25.8 ± 5	56.1 ± 4	49.2 ± 7	27.1 ± 9	72.1 ± 31	202.8 ± 80	77.9 ± 29	36.6 ± 8	24.6 ± 6	21.8 ± 7	41.4 ± 8
**MIP‐2**	47.8 ± 7	40.7 ± 14	**120.1 ± 10**	**83.8 ± 10**	53.7 ± 15	35.1 ± 11	103.6 ± 38	56.6 ± 12	74.7 ± 12	52.1 ± 10	71.3 ± 16	90.4 ± 12
**RANTES**	35.8 ± 5	33.0 ± 4	**71.3 ± 8**	**46.4 ± 4**	14.3 ± 2	13.5 ± 3	8.7 ± 2	13.5 ± 3	38.2 ± 4	29.7 ± 2	38.3 ± 4	28.2 ± 6
**TNF‐α**	3.4 ± 0.4	2.9 ± 1	5.3 ± 0.4	6.2 ± 0.4	3.1 ± 1	3.9 ± 1	7.1 ± 4	5.9 ± 2	**3.8 ± 0.1**	**8.4 ± 1**	**3.7 ± 1**	**12.0 ± 3**
**VEGF**	0.4 ± 0.03	0.4 ± 0.04	9.0 ± 3	3.1 ± 1	0.3 ± 0.02	0.4 ± 0.03	0.5 ± 0.1	0.4 ± 0.04	0.7 ± 0.1	0.5 ± 0.05	0.4 ± 0.03	0.6 ± 0.1

Unpaired, parametric *t*‐tests were performed within sex and model, between no cancer (PBS/WT) and cancer (LLC, C26, and APC) groups for the concentrations of each cytokine. Numbers in bold, within sex and within model, are significantly different (*P *< 0.05) between non‐cancer and cancer groups. Values expressed as means ± SD.

Within the LLC model, eotaxin, IFNγ, IL‐2, IL‐5, IL‐12p40, IL‐13, interferon γ‐induced protein 10 (IP‐10; CXCL10), monokine induced by gamma interferon (MIG; CXCL9), macrophage inflammatory protein‐2 (MIP‐2; CXCL2) and RANTES (regulated on activation, normal T cell expressed and secreted) were all lower in female tumour bearing animals compared with PBS controls (*P* = 0.0016, 0.0292, 0.0007, 0.0182, 0.0173, <0.0001, 0.0041, <0.0001, 0.0331 and 0.0236, respectively), but were not different between PBS and cancer in the male groups. IL‐1α was elevated in tumour bearing females (*P* = 0.0016), but not different in male groups, while granulocyte colony‐stimulating factor (G‐CSF) was higher in both male and female tumour bearing groups compared with PBS controls (*P* = 0.0188 and <0.0001, respectively).

Furthermore, in the C26 model, IL‐1α was only elevated in the male tumour bearing group (*P* = 0.0326), whereas G‐CSF, IL‐6, IP‐10 and keratinocyte‐derived chemokine (KC; CXCL1) were higher in male (*P* = 0.0021, 0.0056, <0.0001 and 0.0085, respectively) and female tumour bearing groups (*P* = 0.0045, 0.0416, <0.0001 and 0.0165, respectively) compared with male and female PBS controls.

Finally, within the APC model, eotaxin was lower in male (*P* = 0.0010) and female (*P* = 0.0204) tumour bearing groups. G‐CSF, IL‐6, and TNF‐α were higher in male (*P* = 0.0045, 0.0019, 0.0022, respectively) and female (*P* = 0.0139, 0.0018, 0.0179, respectively) tumour bearing groups compared with WT. IL‐1β and IL‐17 were both elevated in female tumour bearing groups, but unchanged in males (*P* = 0.0102 and 0.0004, respectively) compared with WT. Finally, IL‐10 was higher in male tumour bearing groups (*P* = 0.0007), while IL‐13 and MIG were lower in male tumour bearing groups (*P* = 0.0012 and 0.0038, respectively). Cytokines IL‐3, IL‐4, IL‐7, IL‐9, IL‐12p70, IL‐15, lipopolysaccharide‐induced CXC chemokine (LIX; CXCL5), macrophage colony‐stimulating factor (MCSF), macrophage inflammatory protein‐1α (MIP‐1α; CCL3) and vascular endothelial growth factor (VEGF) was not statistically different between models.

### 3.3 Sex‐dependent inflammatory responses in circulating cytokines in LLC cachexia

Analyses of circulating cytokine level in both sexes in the LLC model were performed to examine potential sex differences within the LLC model of cachexia. Figure 2a illustrates the relative expression (fold‐change; cancer vs. sex‐matched control) of all 29 cytokines. Fold‐change was chosen to enable the visual and descriptive comparisons of the inflammatory response to tumour burden between sexes and groups without being influenced by baseline differences in sex or mouse background in the circulating levels of these targets. Of the 29 cytokines, four pro‐inflammatory (IL‐2, IFNγ, IL‐1α, IL‐6), five pro‐inflammatory chemokines (leukaemia inhibitory factor [LIF], eotaxin, RANTES, KC, G‐CSF) and four anti‐inflammatory cytokines/chemokines (IL‐13, MIG, IP‐10 and MIP‐2) were significantly different from the PBS group in one or both sexes (Figure 2a, Table 2; *P* < 0.05).

To formally assess biological sex differences and cancer effects, two‐way ANOVA was performed on raw cytokine concentrations comparing male and female LLC animals with their sex‐matched controls (Figure 2a; Table 2). There was a main effect of sex in IL‐3 (*P* = 0.0001), IL‐15 (*P* = 0.002), VEGF (*P* < 0.0001), IL‐6 (*P* = 0.01), RANTES (*P* = 0.0004), KC (*P* = 0.0001), IL‐10 (*P* = 0.0001) and MIP‐1α (*P* < 0.0001), suggesting baseline levels of these cytokines/chemokines may differ between sexes (Figure 2a). The main effect of cancer was noted in RANTES (*P* = 0.03) and KC (*P* = 0.01) (Figure 2a). Interactions between cancer and sex were found in IL‐2 (*P* = 0.006), IFNγ (*P* = 0.03), IL‐5 (*P* = 0.04), IL‐1α (*P* = 0.001), IL‐13 (*P* = 0.0002), IL‐4 (*P *< 0.0001), MIG (*P* < 0.0001) and IP‐10 (*P* = 0.0004), suggesting the inflammatory response of these targets to tumour burden may be sex‐dependent (Figure 2a).

Notably, while we note elevated IL‐6, which is considered a hallmark inflammatory cytokine in cancer cachexia (Puppa et al., 2014; White et al., 2013; White et al., 2012), it was only significant in male, but not female, LLC tumour‐bearing animals (Figure 2a,b; Table 2). In contrast, G‐CSF was significantly elevated in the circulation of both male and female tumour‐bearing animals (Figure 2a,c; Table 2), while the anti‐inflammatory cytokine IL‐13 was significantly lower only in female tumour‐bearing animals (Figure 2a,d; Table 2).

### 3.4 Immune imbalance between pro‐ and anti‐inflammatory cytokines as a strong predictor of TA wasting in female LLC

We performed correlation analyses to identify cytokine signals that showed consistent relationships with muscle mass across multiple cachexia models and across sex‐stratified and combined analyses (Supporting information File 2). Unbiased correlation screening revealed a robust and recurrent pattern in which inflammatory and myeloid‐associated cytokines were negatively associated with tibialis anterior (TA) muscle mass. Among these, G‐CSF emerged as the most consistent correlate across all three cancer cachexia models examined (LLC, C26 and APC), demonstrating strong negative associations with TA mass in both sexes (Supporting information File 2).

As established reference points, we additionally examined TNF‐α and IL‐6 due to their well‐described roles as pro‐inflammatory mediators in cachexia (Puppa et al., 2014; White et al., 2013; White et al., 2012). In contrast to these pro‐inflammatory signals, IL‐13 showed positive associations with TA mass in females across multiple models, emerging as a potential protective cytokine. This observation motivated further exploration of the balance between pro‐ and anti‐inflammatory signalling rather than isolated cytokine levels alone.

To capture this immune balance, we calculated ratios between key pro‐inflammatory cytokines (IL‐6 and G‐CSF) and anti‐inflammatory signals, which strengthened several associations with TA mass and provided additional insight into the immune remodelling accompanying cachexia progression (Supporting information File 2). TA muscle mass was selected as the primary outcome variable because it exhibited the most consistent reductions across tumour‐bearing animals of different models and sexes (Table 1), despite normalized TA mass in LLC males within this subset not differing from controls.

Interestingly, no correlations were found between IL‐6 or TNF‐α levels and percentage change in TA weight for LLC males or females (Figure 2e, Supporting information File 2). However, when sexes were combined, there was a negative association between IL‐6 levels and TA weight (*r* = −0.5, *P* = 0.007, Supporting information File 2). In males, G‐CSF showed no correlation with TA mass; however, in females a moderate negative correlation (*r* = −0.65; *P* = 0.008) was observed (Figure 2f). IL‐13 was a better predictor of muscle mass than IL‐6 or G‐CSF, showing a strong correlation in females (*r* = 0.77; *P* = 0.005) (Figure 2g). Considering this, we examined the ratio between pro‐inflammatory and anti‐inflammatory markers in correlation with TA mass. This ratio was selected to reflect the overall imbalance in the inflammatory milieu, which may more accurately predict muscle mass loss than isolated systemic increases in pro‐inflammatory markers or reductions in anti‐inflammatory responses alone. In males, we did not observe a significant correlation between G‐CSF/IL‐13 ratio and TA mass, while a strong correlation (*r* = −0.77; *P* = 0.005) was noted in females, suggesting this ratio may represent a novel, reliable correlation to predict LLC‐induced TA mass loss in females (Figure 2h).

To facilitate broader exploration of cytokine–phenotype relationships beyond the focused analyses presented here, we developed an interactive Shiny application that allows dynamic visualization of correlations across cytokines, phenotypic outcomes, sex and cancer models. The CytokineExplorer application enables users to interrogate additional phenotype comparisons and alternative correlation models not fully displayed in the main manuscript, providing an expandable resource for hypothesis generation and data transparency (link provided in Methods).

### 3.5 Model‐ and sex‐dependent circulating cytokine responses in colorectal cachexia

Analysis of circulating cytokines in C26 mice were compared with *Apc^Min/+^* to gain insight into circulating cytokines in two different models of colorectal cachexia. Furthermore, we directly compared circulating profiles between males and females to establish whether a sexual dimorphism exists in circulating cytokines within the selected models. Figure 3a illustrates the relative differences of all 29 quantifiable cytokines. Data were expressed as fold change from the healthy control of each model and respective sex, allowing for better interpretation when comparing the response in cytokine expression across cancer models without the influence of baseline control levels. Alternatively, the data can be further explored in its original format using the CytokineExplorer application. Of the 29 cytokines, six proinflammatory cytokines (IL‐1α, IL‐1β, IL‐6, IL‐17 and TNF‐α), three proinflammatory chemokines (eotaxin, G‐CSF and KC), and three anti‐inflammatory cytokines/chemokines (IL‐10, IL‐13 and IP‐10) were significantly different from healthy controls in one or more models/sexes (Figure 3a; Table 2; *P *< 0.05).

Two‐way ANOVAs were subsequently conducted using values from fold change analyses of control versus cancer to rule out baseline differences between the different colorectal cachexia models (i.e., C26 vs. APC). These analyses were performed among cytokines significantly different from their healthy controls in one or more models/sexes (Table 2; *P* < 0.05). Among the pro‐inflammatory cytokines, a main effect of sex was noted for IL‐1α, where it was 1.2‐fold higher in males (*P* = 0.0021), and a main effect of model in IL‐1β and TNF‐α, where they were 1.1‐ and 1.2‐fold higher in APC groups, respectively (*P* ≤ 0.0001 and *P* = 0.0095, respectively) (Figure 3a).

Importantly, an interaction between biological sex and cancer model was noted between groups for IL‐6 and IL‐17. IL‐6 was 12.4‐fold higher in C26 males compared with APC males (*P* = 0.001), and 25‐fold higher in C26 males compared with C26 females (*P* = 0.0004) (Figure 3a,b), while IL‐17 was 10.9‐fold higher in APC males compared with C26 males (*P* ≤ 0.0001), and 3.6‐fold higher in APC males compared with APC females (*P* ≤ 0.0001) (Figure 3a). Within the pro‐inflammatory chemokines, there were main effects on G‐CSF and KC levels, where G‐CSF was 5‐fold higher in APC groups (*P* = 0.0005) (Figure 3a,c), but KC was 3.2‐fold higher in C26 groups (*P* = 0.0005) (Figure 3a). There was an interaction between biological sex and cancer model for eotaxin levels, where they were 0.8‐fold higher in C26 females compared with C26 males (*P* = 0.0042) and 2‐fold higher in C26 females compared with APC females (*P* ≤ 0.0001) (Figure 3a).

Finally, within the anti‐inflammatory cytokines/chemokines, there was a main effect of sex on IL‐13 levels, where they were 0.5‐fold higher in male groups (*P* = 0.0003) (Figure 3a,d). Furthermore, there were interactions between biological sex and cancer model on IL‐10 and IP‐10 levels, where IL‐10 was 12.8‐fold higher in APC males compared with C26 males (*P* ≤ 0.0001), 1.5‐fold higher in APC males compared with APC females (*P* ≤ 0.0001) and 20.2‐fold higher in C26 females compared with APC females (*P* = 0.0176) (Figure 3a), while IP‐10 was 2.6‐fold higher in C26 males compared with APC males (*P* ≤ 0.0001), 0.4‐fold higher in C26 males compared C26 females (*P* = 0.0398) and 1.2‐fold higher in C26 females compared with APC females (*P* = 0.0085) (Figure 3a).

### 3.6 Immune imbalance between pro‐ and anti‐inflammatory cytokines as a strong sex‐unbiased predictor of TA wasting in colorectal cancers

We conducted further correlation analyses with the same rationale as for the LLC model (Figure 3e–h). In C26 females, a negative association between IL‐6 levels and TA mass was noted (Figure 3e; *r* = −0.61, *P* = 0.04). C26 females displayed no significant other correlations of TNF‐α, IL‐13, G‐CSF, or G‐CSF/IL‐13 with TA weight (Figure 3e–h, Appendix Figure A1). In contrast, in C26 males, IL‐6 (Figure 3e; *r* = −0.72, *P* = 0.003), G‐CSF (Figure 3f; *r* = −0.56; *P* = 0.03) and G‐CSF/IL‐13 (Figure 3l; *r* = −0.57; *P* = 0.02) were negatively correlated with TA weight. No associations between TNF‐α or IL‐13 were noted in C26 males. In APC females, TNF‐α (Figure A1; *r* = −0.8, *P* = 0.0003), IL‐6 (Figure 3e; *r* = −0.77; *P* = 0.0008), G‐CSF (Figure 3f; *r* = −0.87; *P* = 2.7^−5^), IL‐13 (Figure 3g; *r* = 0.63, *P* = 0.01) and G‐CSF/IL‐13 (Figure 3h; *r* = −0.82; *P* = 0.001) were correlated with TA weight. Finally, in APC males TNF‐α (Figure A1; *r* = −0.66, *P* = 0.007), IL‐6 (Figure 3e; *r* = −0.59; *P* = 0.03), G‐CSF (Figure 3f; *r* = −0.85; *P* = 0.0001) and G‐CSF/IL‐13 (Figure 3h; *r* = −0.82; *P* = 0.0001) were negatively correlated with TA weight.

**FIGURE 1:**
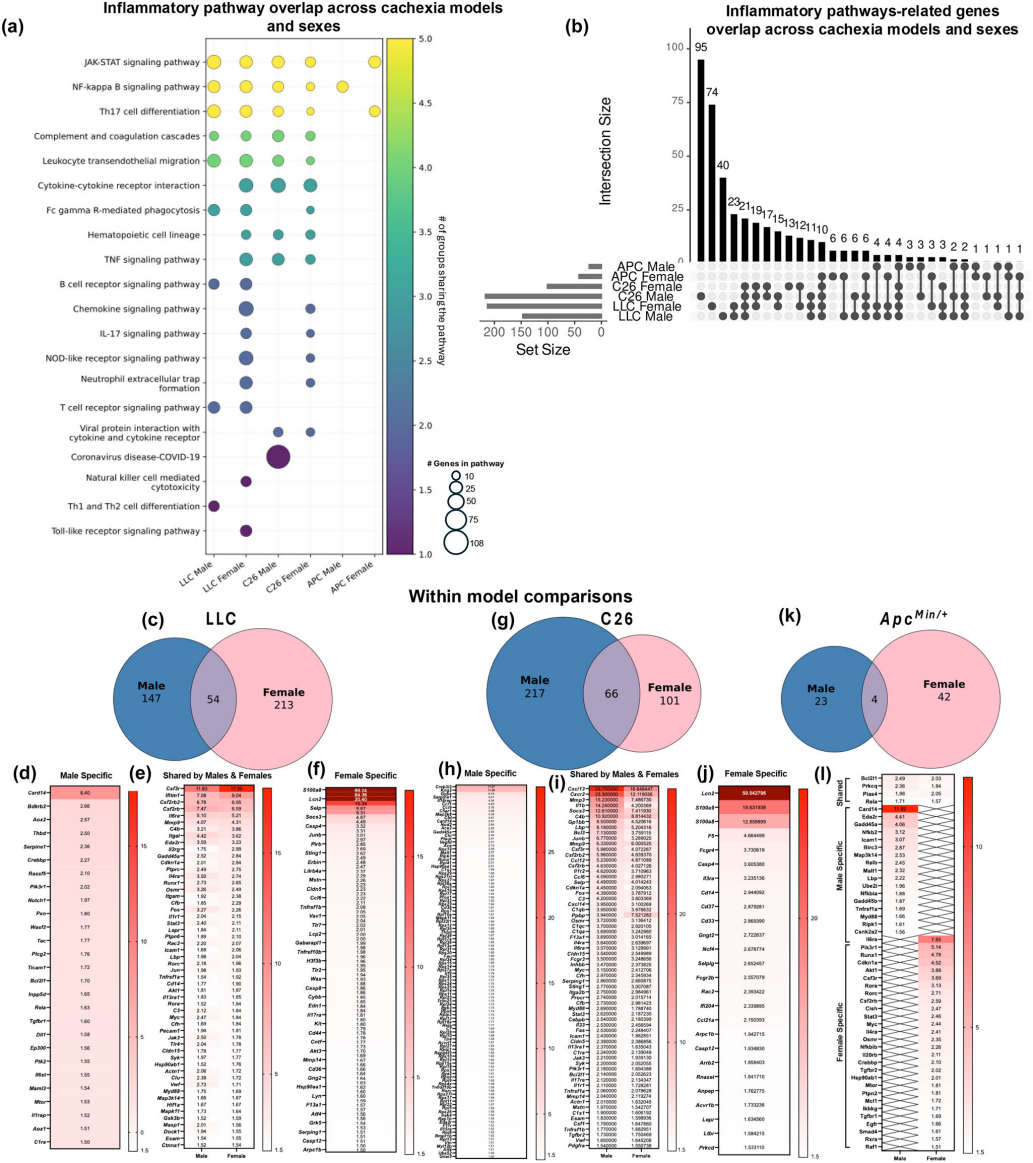
RNA‐sequencing data filtered for immune systems and inflammatory pathways utilizing KEGG pathways definitions enriched in skeletal muscle undergoing CC. KEGG inflammatory pathways identified and number of genes within each pathway (a). Upset plot for inflammatory‐associated genes across all groups and sexes (b). The number of differentially expressed genes (DEGs) for males and females LLC (c) and fold change in gene expression of male‐specific DEGS (d), sex‐shared DEGs (e), and female‐only DEGs (f). Number of DEGs for males and females C26 (g) and fold change in gene expression of male‐specific DEGS (h), sex‐shared DEGs (i), and female‐only DEGs (j). The number of DEGs for males and females ApcMin/+ (k) and fold change in gene expression of male‐specific DEGS, sex‐shared DEGs, and female‐only DEGs (l). Significance considered at adj‐*P*‐value < 0.05.

**FIGURE 2:**
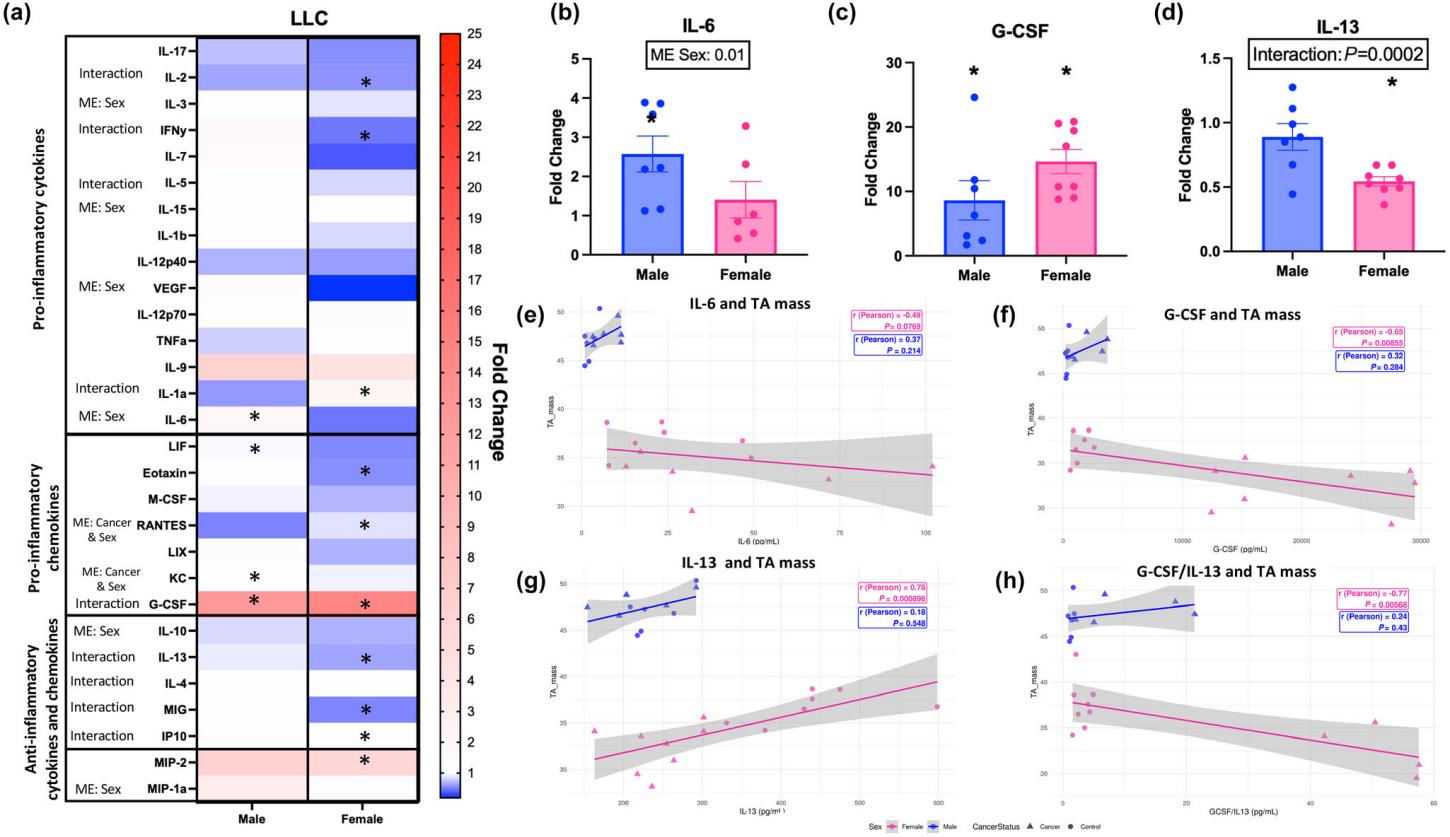
LLC cytokine/chemokine expression. Fold change (cancer vs control) in cytokine heatmap (a). 2‐way‐ANOVA results are denoted as text, and T‐text within sex significance is denoted as asterisks.Males and females circulating concentration (pg/mL) of IL‐6 (b), G‐CSF (c), and IL‐13 (d). Males and Females correlation between tibialis anterior (TA) and levels of IL‐6 (e), G‐CSF (f), IL‐13 (g), and G‐CSF/IL‐13 (h). n = 6–8/group. Data expressed as mean +/− SD, with significant alpha value set at 0.05. “Interaction” indicates interaction of model and sex, “ME: Sex” indicates main effect of sex, and “ME: Model” indicates main effect of model.

**FIGURE 3:**
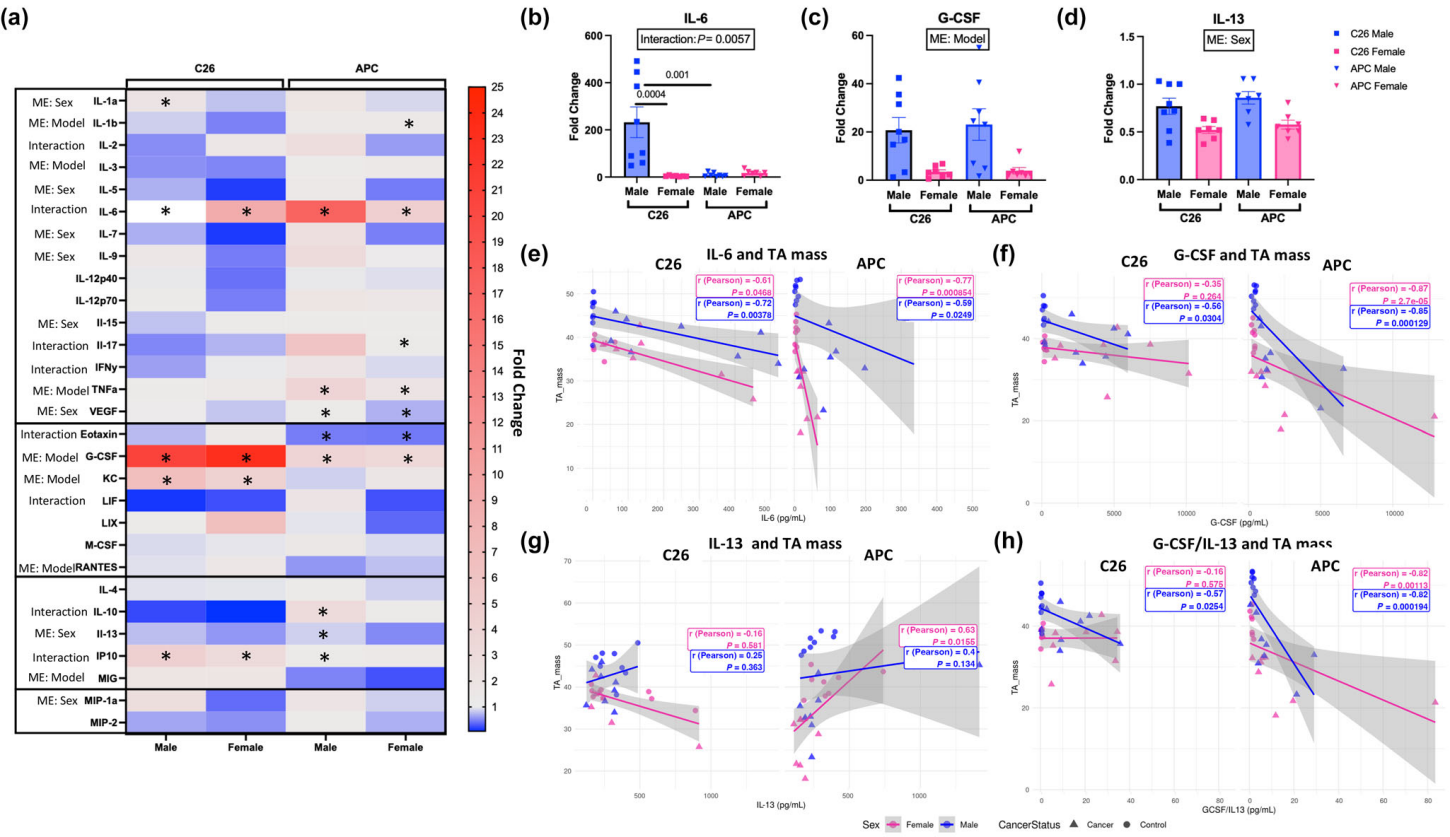
Comparison of cytokine levels in both models of colorectal cancer cachexia. Fold change (cancer vs control) in cytokine heatmap (a). 2‐way‐ANOVA results are denoted as text, and T‐text within sex significance is denoted as asterisks.C26 and APC males and females circulating concentration (pg/mL) of IL‐6 (b), G‐CSF (c), and IL‐13 (d). C26 and APC males and Females correlation between tibialis anterior (TA) and levels of IL‐6 (e), G‐CSF (f), IL‐13 (g), and G‐CSF/IL‐13 (h). n = 7–8/group. Data expressed as mean +/− SD, with significant alpha value set at 0.05. “Interaction” indicates interaction of model and sex, “ME: Sex” indicates main effect of sex, and “ME: Model” indicates main effect of model.

### 3.7 Collapsed LLC, C26 and APC male and female models demonstrate negative correlations with TA muscle mass and G‐CSF cytokine concentrations

To further explore potential sources of homogeneity across cachexia models (LLC, C26 and APC), we conducted correlation analyses of TNF‐α, IL‐6, G‐CSF, IL‐13 and the G‐CSF/IL‐13 ratio with TA mass by collapsing data within each sex across all three models. In both males and females, TNF‐α was negatively correlated with TA mass (Figure 4a). In males, IL‐6 (Figure 4b; *r* = −0.48, *P* = 0.001), while in females G‐CSF (Figure 4c; *r* = −0.42, *P* = 0.0034), and IL‐13 (Figure 4c; *r* = 0.33, *P* = 0.03), was associated with TA mass. The ratio between G‐CSF/IL‐13 and TA mass approached significance in combined models of males (Figure 4d; *r* = −0.28, *P* = 0.07) and females (Figure 4d; *r* = −0.3, *P* = 0.06).

**FIGURE 4:**
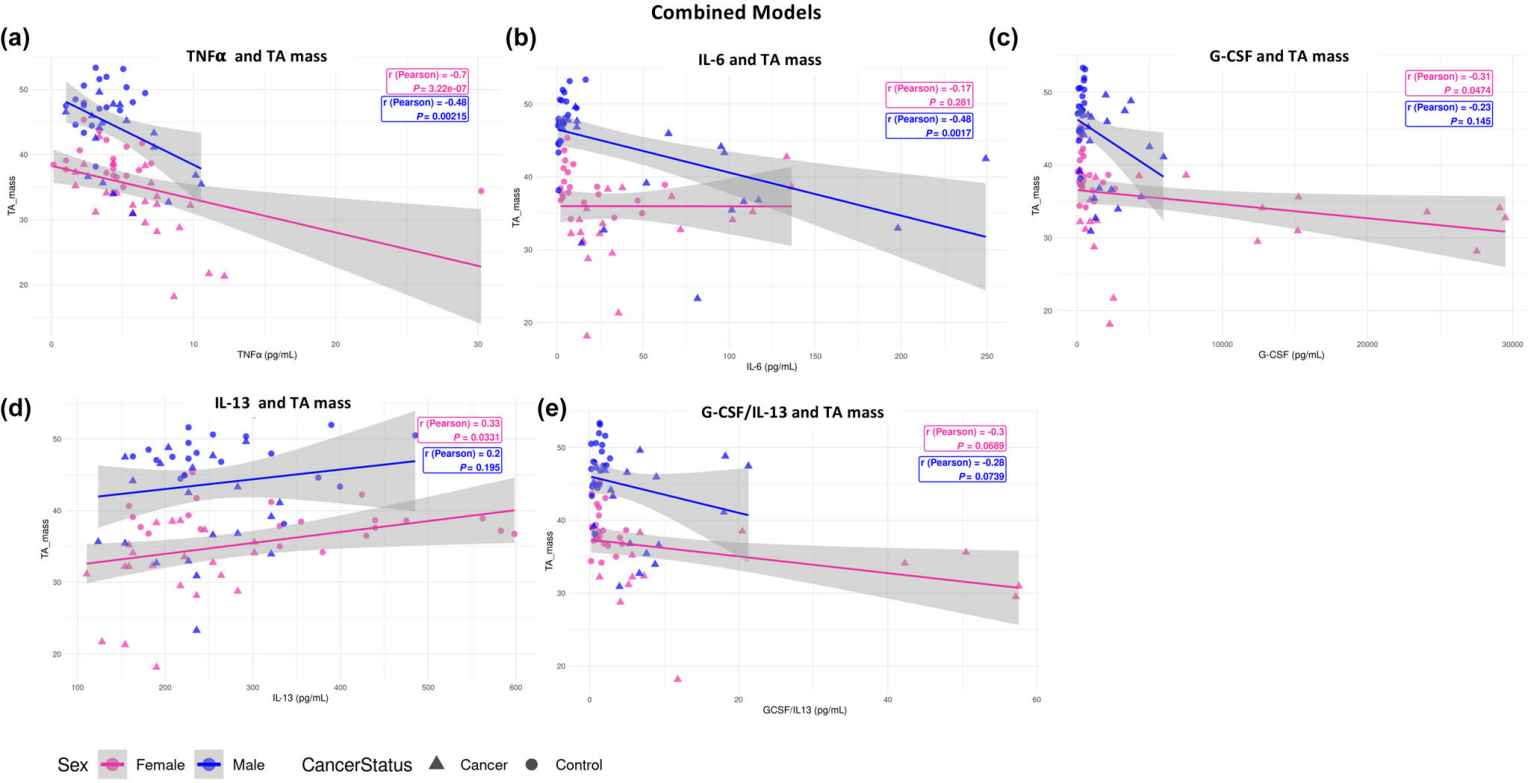
Correlation analyses of cytokines with tibialis anterior (TA) muscle mass combining all three cachexia models (LLC, C26, ApcMin/+) and healthy controls, stratified by sex. (AD) Male‐ and female‐specific correlations associations between TA muscle mass and TNF‐α (a), IL‐6 (b), G‐CSF (c), and IL‐13 (d), G‐CSF/IL‐13 (e). n = 7–8/group.

When biological sexes were combined, the two stronger negative correlations between TA and cytokine level emerged as TNF‐α (Figure 5a, *r* = −0.49, *P* = 4.2^−6^), and IL‐6 (Figure 5c, *r* = −0.37, *P* = 0004). Although diluted, negative correlations were maintained between TA mass and G‐CSF (Figure 5c, *r* = −0.25, *P* = 0.02), as well as with G‐CSF/IL‐13 ratio (Figure 5e, *r* = −0.32, *P* = 003), while the positive association between IL‐13 levels and TA mass was lost (Figure 5d).

**FIGURE 5:**
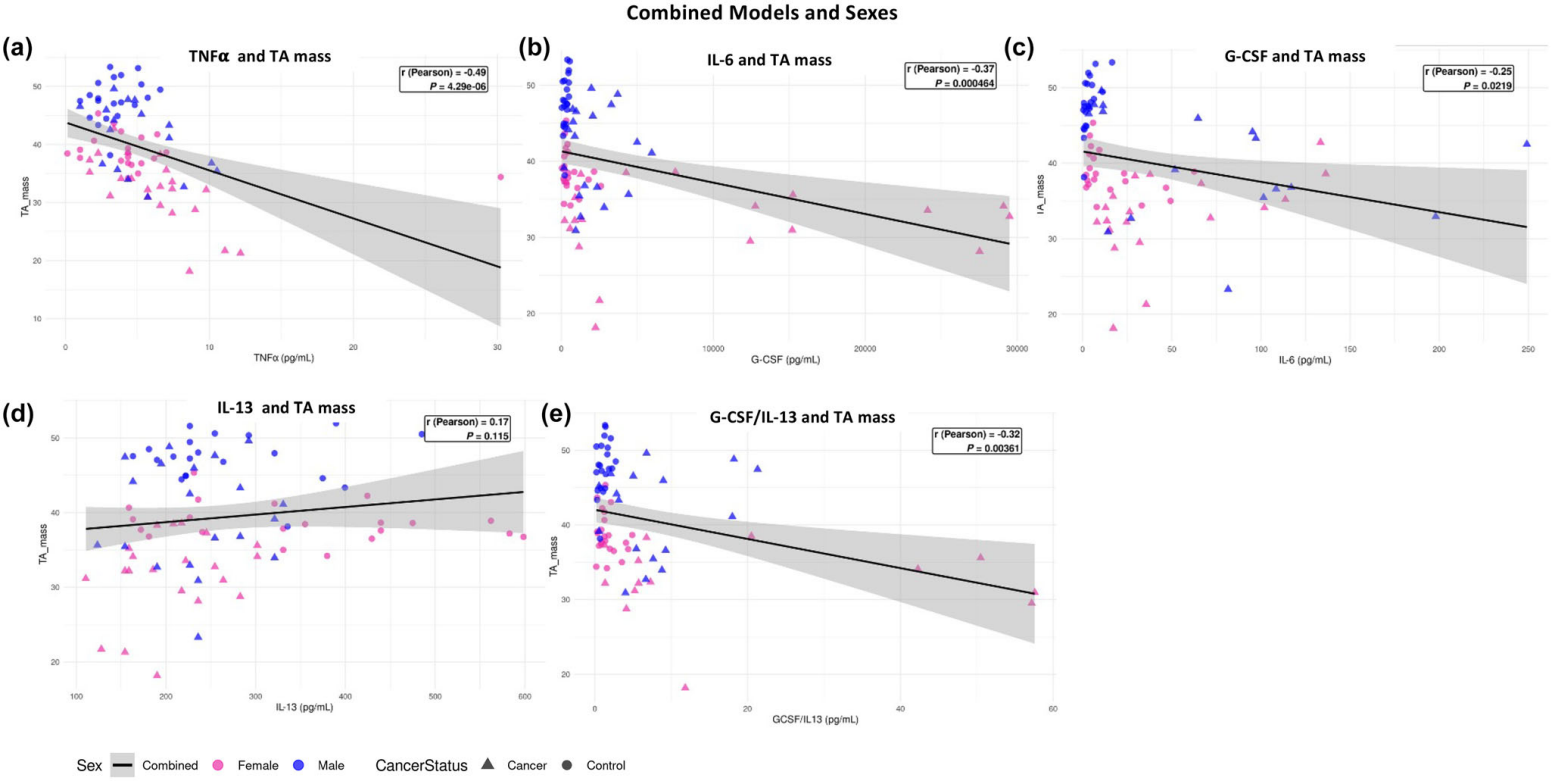
Correlation analyses of cytokines with tibialis anterior (TA) muscle mass combining both sexes and all three cachexia models (LLC, C26, ApcMin/+) and healthy controls. (AD) Male‐ and female‐specific correlations associations between TA muscle mass and TNF‐α (a), IL‐6 (b), G‐CSF (c), and IL‐13 (d), G‐CSF/IL‐13 (e). n = 7–8/group.

## 4 DISCUSSION

Cachexia presents a substantial challenge to effective cancer management due to its devastating impact on patient outcomes (Argilés et al., 2015; Fearon et al., 2013). One of the primary obstacles to treating cachexia is its frequent late‐stage diagnosis, at which point patients are often too frail to tolerate anti‐cancer therapies (Argilés et al., 2015; Fearon et al., 2013). This underscores the critical importance of early identification and intervention. Inflammatory markers have been widely investigated for their role in mediating skeletal muscle mass loss and dysfunction, hallmarks of cachexia. Numerous studies have implicated cytokines and chemokines such as IL‐6, IL‐1, IFN‐γ, VEGF‐A and TNF‐α as key drivers of cachectic pathology. Importantly, the necessity of these cytokines for the development of cachexia appears to exhibit sex‐specific biases, with males more consistently exhibiting dependence on these pathways for cachectic development than females (Carson & Baltgalvis, 2010; Hetzler et al., 2015; Hetzler et al., 2017; Zhong et al., 2022).

Our study aimed to assess cytokine/chemokine profiles of distinct preclinical cachexia models. We demonstrated consistent upregulation of G‐CSF, a pro‐inflammatory cytokine, across both sexes and independent of cancer type. Elevated G‐CSF levels demonstrated a robust correlation with TA muscle loss, particularly when analysed in ratio with the anti‐inflammatory cytokine IL‐13. Examination of cytokine ratios provides insightful information of the inflammatory environment within cancer cachexia and may help to expand on the relationships between pro‐ and anti‐inflammatory cytokines. Our findings indicate the ratio of G‐CSF to IL‐13 may be a reliable global marker of cachexia, suggesting potential utility in preclinical research and clinical applications. Importantly, several cytokine–phenotype relationships which were strong within sex‐specific analyses became attenuated or lost when data were combined across sexes or models, indicating that biologically meaningful signals can be diluted when these sources of heterogeneity are ignored. This observation reinforces the need to consider sex and model context when interpreting inflammatory biomarkers in cachexia.

G‐CSF is well‐recognized for its role in neutrophil maturation and mobilization from the bone marrow (Semerad et al., 2002). Its expression is typically induced by inflammatory signals such as those stemming from TNF‐α and IL‐1β (Martin et al., 2021). Upon binding its receptor, G‐CSF activates key transcription factors, including STAT1, STAT3 and STAT5, which mediate downstream signalling through the JAK/STAT, phosphoinositide 3‐kinase (PI3K)/protein kinase B (AKT), and mitogen‐activated protein kinase (MAPK)/extracellular signal‐regulated kinase (ERK) pathways (Tian et al., 1996). These cascades can culminate in inflammatory transcriptional program activation, including NF‐κB activation and neutrophil mobilization (Karagiannidis et al., 2021). While transient G‐CSF elevation has been implicated in promoting muscle regeneration post‐injury by enhancing myocyte proliferation (Hara et al., 2011), sustained upregulation of G‐CSF in cancer‐associated cachexia can contribute to chronic inflammatory remodelling and impaired skeletal muscle homeostasis. In the context of cachexia, chronic G‐CSF elevation may be associated with persistent inflammatory signalling, including the activation of JAK/STAT‐related pathway. Circulating G‐CSF levels were notably elevated across the preclinical models used in this study, which displayed a range of cachexia severities from mild (LLC and C26) to severe (*Apc^Min/+^*). In our data set, we showed that the *Csf3r* gene, which encodes for the G‐CSF receptor, was the most upregulated inflammation‐related gene in skeletal muscle of both sexes of the LLC and C26 models, as well as in female APC, while JAK/STAT and NF‐κB‐associated genes were upregulated in both sexes of all three models explored here. However, because these pathways can be activated by multiple inflammatory mediators, including IL‐6 and TNF‐α, the present data do not establish G‐CSF as the sole driver of these transcriptional responses. These data instead suggest that elevated G‐CSF occurs alongside a broader conserved inflammatory signalling profile in cancer cachexia and may represent a candidate biomarker associated with cachexia progression across models and sexes.

Consistent with our findings, elevated G‐CSF levels have been observed in multiple clinical and preclinical models of cachexia (Bahar et al., 2010; Bordbar et al., 2012; Matsuyama et al., 2015; Mroczko et al., 2006), with cachectic patients exhibiting higher G‐CSF levels than weight‐stable cancer patients at similar disease stages (Costa et al., 2019) suggesting the translational relevance of the findings here. Conversely, in models of muscle dystrophy, such as the *mdx* mouse, short (2 days) and long‐term (2 weeks) G‐CSF administration therapies (i.e., daily intraperitoneal injections of 5 µg G‐CSF diluted in saline) have shown beneficial effects, including improved survival and reduced muscle atrophy and dysfunction (Hayashiji et al., 2015). These outcomes are attributed to G‐CSF's role in satellite cell expansion and enhanced muscle regeneration, which counteracts degeneration in dystrophic conditions. However, these regenerative effects are distinct from the pathophysiological mechanisms driving cachexia as these treatments would allow a regular pulsatile increase in G‐CSF and its downstream signalling in contrast to a more truly chronic elevation in the pathology of cachexia.

In addition to heightened circulation of pro‐inflammatory cytokines, cachexia is often characterized by a suppression of anti‐inflammatory factors, including IL‐4, IL‐10 and IL‐13, leading to a systemic inflammatory imbalance (Molanouri Shamsi et al., 2017). The ratio of pro‐ to anti‐inflammatory cytokines has thus garnered interest as a potential marker of disease severity (Molanouri Shamsi et al., 2017). IL‐13, in particular, has been shown to suppress pro‐inflammatory cytokines including IL‐1β, IL‐6 and TNF‐α, and has been described as an anti‐cachectic cytokine (Argilés et al., 2003; Koj, 1998). Although its role in muscle loss during cachexia remains underexplored, IL‐13 has demonstrated protective effects in sepsis‐induced atrophy models, where recombinant IL‐13 treatment attenuates muscle wasting (Akama et al., 2024). In our study, circulating IL‐13 levels were significantly reduced in LLC females and *Apc^Min/+^* males though mean reductions appeared consistently across models and sexes.

Several cytokine–phenotype relationships that were strong within sex‐specific analyses became attenuated or lost when data were combined across sexes or models, indicating that biologically meaningful signals may be diluted when such heterogeneity is ignored. This observation reinforces the need to consider sex and model context when interpreting inflammatory biomarkers in cachexia. Notably, even after combining models and sexes, the correlation between G‐CSF and IL‐13 levels and TA mass persisted, where higher G‐CSF/IL‐13 ratios were correlated with lower TA masses. This finding supports the potential of the G‐CSF/IL‐13 ratio as a biomarker of cachexia, capable of capturing systemic inflammatory dysregulation in a variety of preclinical cancer models.

### 4.1 Conclusion

The concept of circulating pro‐inflammatory factors interacting with peripheral organs to drive cachexia is well‐established. In this study, we demonstrated that numerous genes encoding cytokines and their receptors are upregulated in cachectic skeletal muscle, underscoring the muscle's active role in responding to systemic inflammation. We further showed that circulating levels of pro‐inflammatory cytokines are elevated in both males and females across all three models of cachexia studied. Among these, G‐CSF emerged as a consistently upregulated cytokine in all models and sexes, positioning it as a potentially robust and reliable biomarker for predicting muscle mass loss in cancer, irrespective of sex. Here we create CytokineExplorer ShinyApp, a new interactive resource tool that researchers can utilize to explore multiple cytokine‐phenotype relationships, promoting an open platform for hypothesis generation.

As a model, we propose secreted factors, such as IL‐6 and G‐CSF, engage in crosstalk with skeletal muscle, triggering signalling cascades promoting pro‐atrophic gene programs while suppressing the expression of anti‐inflammatory cytokines, such as IL‐10 and IL‐13, and their downstream signalling pathways (Figure 6). This disruption exacerbates systemic inflammation, ultimately driving the muscle‐wasting phenotype characteristics of cachexia. While we relied on robust pre‐clinical cancer models and tools to assess cytokine levels, while employing careful interpretation, the findings within this study rely on exploratory measurements and associative conclusion driven by correlation analysis in relatively pre‐clinical cancer cohorts. To further validate our observations, future studies should focus on confirming these markers' correlations with skeletal muscle mass and function to develop a reliable biomarker for the early detection and management of cachexia.

**FIGURE 6:**
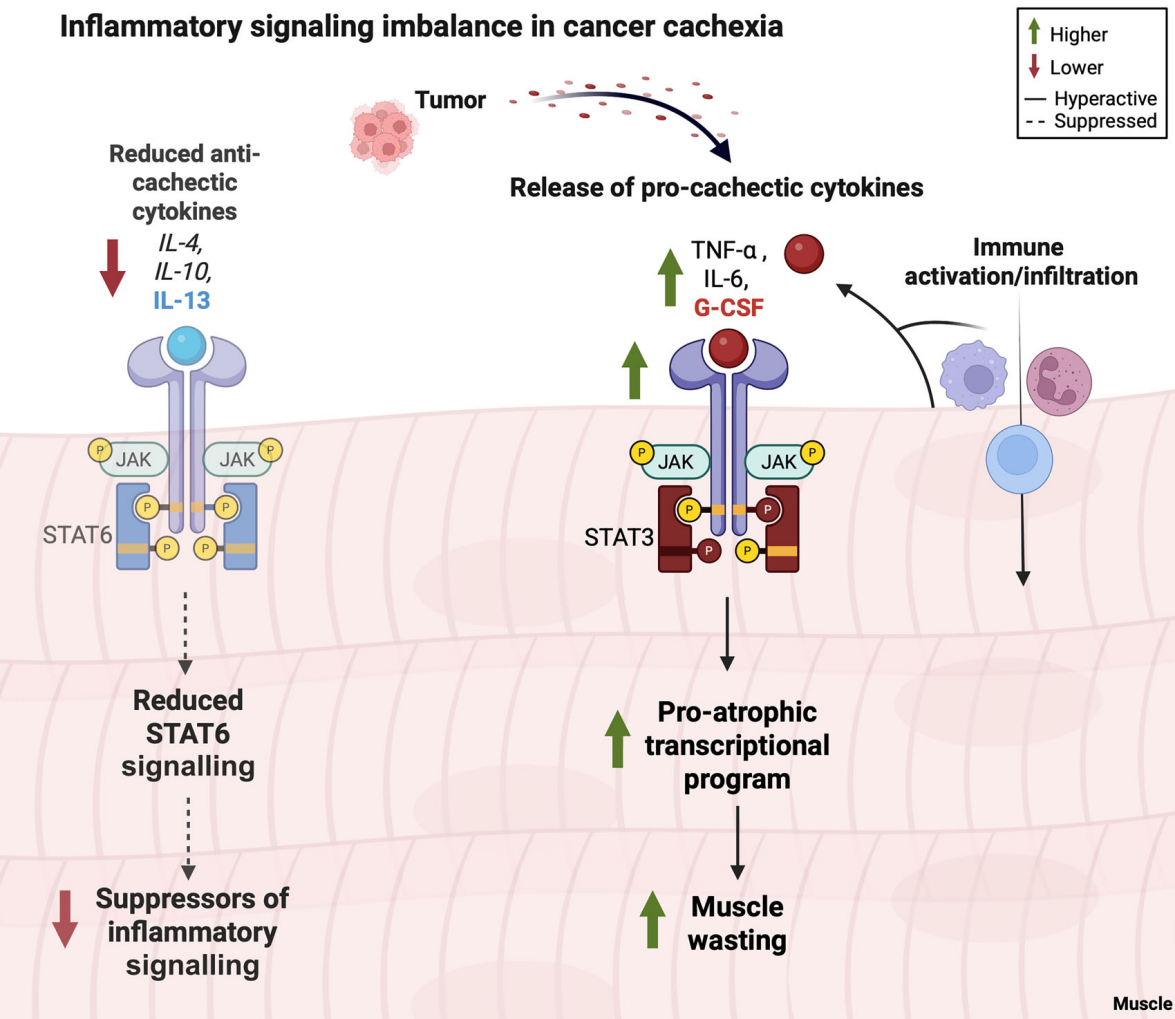
Schematic summary illustrating the proposed mechanism: Tumor‐ and infiltrating immune cells releases pro‐inflammatory cytokines, such as TNF‐α, IL‐6 and G‐CSF, activate an inflammatory transcriptional cascade in skeletal muscle, leading to upregulation of pro‐inflammatory signaling and pro‐atrophic activation. Concurrently, this signaling suppresses anti‐inflammatory cytokines (e.g., IL‐10 and IL‐13), failing to counterbalance inflammation and exacerbating muscle atrophy.

## AUTHOR CONTRIBUTIONS

Both first authors Francielly Morena and Eleanor R. Schrems contributed to the study conceptualization, sample collections, sample processing, data collection, data analysis, graphical representations, and manuscript writing. Ana Regina Cabrera was responsible for data organization and correlation analyses and RT aided in this process. Ruqaiza Muhyudin and Sepideh Shakeri aided in the manuscript drafting and data interpretation. Mark Viggars provided support for the creation and maintenance of the Shinny application. Tyrone A. Washington and Nicholas P. Greene were responsible for study conceptualization, supervision, resources, and funding support. All authors have read and approved the final version of this manuscript and agree to be accountable for all aspects of the work in ensuring that questions related to the accuracy or integrity of any part of the work are appropriately investigated and resolved. All persons designated as authors qualify for authorship, and all those who qualify for authorship are listed.

## CONFLICT OF INTEREST

The authors of this manuscript declare no conflicts of interest.

## GENERATIVE AI STATEMENT

The authors confirm that no artificial intelligence tools, including large language models (LLMs), were used in the drafting or revision of this manuscript. All content was conceived, written, and approved solely by the authors.

## Supporting information




**File 1**: RNA‐Sequencing Data output. List of KEGG pathways under inflammation and immune systems. List of filtered differentially expressed genes (DEGs) of each model and sex implicated in any of these inflammatory‐associated pathways.

**File 2**: Correlation analysis of each cytokine concentration and tibialis anterior mass for all cachexia models in both sexes.

## Figures and Tables

**FIGURE A1:**
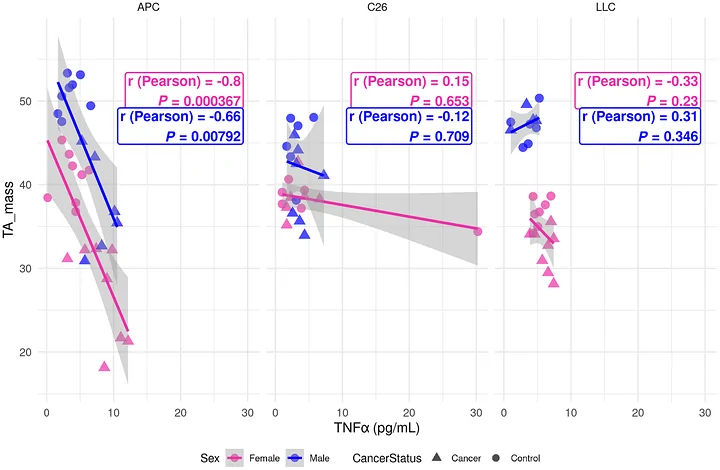
Correlation analyses of cytokines with tibialis anterior (TA) muscle mass for both sexes and combining all three cachexia models (LLC, C26, ApcMin/+) and healthy controls. Male‐ and female‐specific correlations associations between TA muscle mass and TNF‐α stratified by models. *n* = 7–8/group.

## Data Availability

RNA‐sequencing datasets analysed in this study are publicly available in the Gene Expression Omnibus (GEO) under accession numbers GSE114820, GSE222317 and GSE276018. Additional processed data and correlation outputs are available through the CytokineExplorer Shiny application (https://thecachexiatlas.shinyapps.io/CytokineExplorer/) or from the corresponding author upon reasonable request.
